# The potential impact of triage protocols on racial disparities in clinical outcomes among COVID-positive patients in a large academic healthcare system

**DOI:** 10.1371/journal.pone.0256763

**Published:** 2021-09-16

**Authors:** Shireen Roy, Mary Showstark, Benjamin Tolchin, Nitu Kashyap, Jennifer Bonito, Michelle C. Salazar, Jennifer L. Herbst, Katherine A. Nash, Max Jordan Nguemeni Tiako, Karen Jubanyik, Nancy Kim, Deron Galusha, Karen H. Wang, Carol Oladele

**Affiliations:** 1 Department of Chronic Disease Epidemiology, Yale School of Public Health, New Haven, CT, United States of America; 2 Yale School of Medicine Physician Assistant Online Program, Yale Institute of Global Health, National Disaster Medical System, New Haven, CT, United States of America; 3 Department of Neurology, Yale School of Medicine, Epilepsy Center of Excellence, VA Connecticut Healthcare System, Yale New Haven Health, New Haven, CT, United States of America; 4 Yale New Haven Health, New Haven, CT, United States of America; 5 Department of Emergency Medicine, Yale School of Medicine, New Haven, CT, United States of America; 6 Department of Surgery, Yale School of Medicine, National Clinician Scholars Program, New Haven, CT, United States of America; 7 Quinnipiac University School of Law, Frank H. Netter, MD, School of Medicine at Quinnipiac University, North Haven, CT, United States of America; 8 Department of Pediatrics, Yale School of Medicine, National Clinician Scholars Program, Yale School of Medicine, New Haven, CT, United States of America; 9 Yale School of Medicine, New Haven, CT, United States of America; 10 Equity Research and Innovation Center, Yale School of Medicine, New Haven, CT, United States of America; Technion - Israel Institute of Technology, ISRAEL

## Abstract

**Background:**

The COVID-19 pandemic has had a devastating impact in the United States, particularly for Black populations, and has heavily burdened the healthcare system. Hospitals have created protocols to allocate limited resources, but there is concern that these protocols will exacerbate disparities. The sequential organ failure assessment (SOFA) score is a tool often used in triage protocols. In these protocols, patients with higher SOFA scores are denied resources based on the assumption that they have worse clinical outcomes. The purpose of this study was to assess whether using SOFA score as a triage tool among COVID-positive patients would exacerbate racial disparities in clinical outcomes.

**Methods:**

We analyzed data from a retrospective cohort of hospitalized COVID-positive patients in the Yale-New Haven Health System. We examined associations between race/ethnicity and peak overall/24-hour SOFA score, in-hospital mortality, and ICU admission. Other predictors of interest were age, sex, primary language, and insurance status. We used one-way ANOVA and chi-square tests to assess differences in SOFA score across racial/ethnic groups and linear and logistic regression to assess differences in clinical outcomes by sociodemographic characteristics.

**Results:**

Our final sample included 2,554 patients. Black patients had higher SOFA scores compared to patients of other races. However, Black patients did not have significantly greater in-hospital mortality or ICU admission compared to patients of other races.

**Conclusion:**

While Black patients in this sample of hospitalized COVID-positive patients had higher SOFA scores compared to patients of other races, this did not translate to higher in-hospital mortality or ICU admission. Results demonstrate that if SOFA score had been used to allocate care, Black COVID patients would have been denied care despite having similar clinical outcomes to white patients. Therefore, using SOFA score to allocate resources has the potential to exacerbate racial inequities by disproportionately denying care to Black patients and should not be used to determine access to care. Healthcare systems must develop and use COVID-19 triage protocols that prioritize equity.

## Introduction

The SARS-CoV-2 pandemic (also known as coronavirus disease 2019, or COVID-19) has had a devastating impact throughout the United States. As of August 3, 2021, the United States accounted for almost 35 million COVID-19 cases and 608,288 deaths of the 4,235,559 COVID-19 deaths worldwide [1]. The pandemic has placed an unprecedented burden on the American healthcare system [2–4]. This has led to the rationing of personal protective equipment (PPE) and ventilators [3]. Many hospitals have created triage protocols from state disaster plans and are operating under crisis standards of care (CSC) to prioritize patients who will receive certain resources.

There are a number of approaches to creating triage protocols. In general, protocols seek to provide “the greatest benefit to the greatest number of individuals while the fewest resources are used” [5]. “Greatest benefit” may refer to short-term or long-term survival, and policymakers may consider age as a proxy of the number of years a person has left to live. They may also use the “fair innings” approach, which considers the life stages a person has left to experience [5, 6]. First come first served protocols are not preferred, as they favor those who can readily access healthcare institutions [7].

Some protocols will score the severity of a patient’s illness or account for the patient’s comorbidities, but these approaches may unjustly deny resources to marginalized populations, who have higher rates of conditions (i.e. hypertension) that are often factored into these scores [5, 8]. One of these measures is the sequential organ failure assessment, or SOFA, score. The SOFA score includes measures of respiratory function (PaO_2_/FiO_2_, on mechanical ventilation), coagulation (platelet count), mean arterial pressure, liver (bilirubin), central nervous system (Glasgow Coma Scale), and renal functioning (creatinine), is well validated in critical care patients, and is already used as a part of some states’ triage protocols [9–16]. In these protocols, patients with higher SOFA scores could be denied care in resource-limited circumstances based on the assumption that they will have worse clinical outcomes compared to patients with lower SOFA scores.

The use of SOFA score in COVID-19 triage protocols varies. Some states used SOFA score in conjunction with age and/or chronic comorbidities, while others (for example, New York) only used the SOFA score (or a corresponding pediatric measure or different measure of acute illness) to determine access to ventilators in crisis situations [14]. At the time of this study, Connecticut did not have a specific COVID triage protocol in place.

The protocol developed for the Yale-New Haven Health (YNHH) system was shared with the Connecticut Hospital Association and Connecticut State Medical Society. This triage protocol considered SOFA score and “survival-limiting chronic comorbidities…that are expected to result in death within one year regardless of medical intervention,” and also prioritized some pregnant patients and “healthcare workers, first responders, or hospital employees with repeated patient contact during the pandemic” [17]. Age was not considered in the YNHH triage protocol. Triage would be conducted by teams consisting of at least one physician and one nurse not involved in direct patient care. The triage teams would be in contact with ICU attending physicians, ethicists on call, and a triage supervisor (administrator). While the YNHH triage protocol was created to allocate intensive care resources in preparation for a surge of COVID-19 cases, it was not activated.

Concerns raised about the use of SOFA score during the H1N1 pandemic and limited validation in sepsis patients led to increased interest in assessing the potential impact of using SOFA in triage protocols [12, 16]. Prior findings suggest that the use of SOFA in triage could lead to the denial of care to critically ill patients during the H1N1 pandemic and that high SOFA scores are not accurate in predicting mortality for many patients [18, 19]. In general, SOFA scores have not been evaluated for use in crisis standards of care.

Importantly, the performance of SOFA score in accurately predicting outcomes among diverse patients has not been determined; SOFA has been validated among predominantly white patients [15, 20, 21]. Black patients as a whole have higher levels of factors that could negatively impact SOFA score, such as high creatinine and high mean arterial pressure, as a result of less access to healthcare and socioeconomic resources [22, 23]. It is important to note that Black patients may have higher SOFA scores not for any intrinsic biological reason, but because they have higher rates of the conditions that are factored into the SOFA score as a result of historical and current inequities [24].

If Black patients have disproportionately higher SOFA scores compared to white patients, they may be denied access to life-saving treatment, even if their higher SOFA scores do not necessarily translate into higher COVID-associated morbidity and mortality. Thus, there is concern that the use of SOFA score in triage may worsen existing disparities in COVID-19 morbidity and mortality. By using SOFA score to determine access to resources, hospital systems risk disproportionately withholding resources from disadvantaged groups and widening disparities in clinical outcomes. The potential for triage protocols to worsen disparities is concerning, given that data have already shown a clear and disproportionate impact of the virus in Black and Latino populations [2, 25–27].

In this study, we investigated whether using SOFA score as a triage tool had the potential to exacerbate disparities in clinical outcomes among hospitalized COVID-19 patients in the YNHH system. We first investigated differences in peak 24-hour SOFA score by race/ethnicity. We then determined whether in-hospital mortality and intensive care unit (ICU) admission varied by race/ethnicity and other patient characteristics. If minority patients had higher peak 24-hour SOFA scores but were not more likely to die in the hospital or be admitted to the ICU, they could be unfairly denied access to resources under triage protocols that favor patients with lower SOFA scores.

## Methods

### Study design and setting

We conducted a retrospective cohort study of hospitalized COVID-positive patients in the Yale-New Haven Health System (YNHH), a large healthcare system with 2,558 total beds and 225 ICU beds which includes hospitals and providers in Connecticut, New York, and Rhode Island. During the pandemic, many non-ICU beds were converted to accommodate COVID-19 patients, and eligibility for receiving intensive care changed throughout the pandemic. Data were extracted from the YNHHS electronic medical record. Our sample included all patients hospitalized between March 29 and July 28, 2020 who had a positive COVID-19 polymerase chain reaction (PCR) test result. We excluded patients who did not have a SOFA score calculated within 24 hours of admission. The study was approved by the Yale School of Medicine Institutional Review Board (IRB) and a waiver of informed consent was granted.

### Independent variables

Our main independent variable was race/ethnicity (white non-Hispanic, Black non-Hispanic, Hispanic all races, other non-Hispanic race, and unknown) as documented in the electronic medical record (EMR). The “other non-Hispanic” category included patients of Asian, American Indian/Alaskan Native, Native Hawaiian, and other non-Hispanic race. The EMR collects race (white, Black or African American, Asian, American Indian/Alaskan Native, Native Hawaiian, other race, and unknown race) and ethnicity (Hispanic or non-Hispanic) separately; we combined them for this analysis. Other independent variables examined were age, sex, primary language (English, Spanish, or other), and insurance status (private, Medicaid, Medicare, Medicaid/Medicare, or none). Outcomes of interest were in-hospital mortality and admission to the ICU. We investigated the Charlson comorbidity index (calculated within 48 hours of admission) as a potential confounder. The Charlson comorbidity index assigns a weight to each of 17 comorbid conditions, including diabetes, acute myocardial infarction, and renal disease, and is a predictor for in-hospital mortality [28].

### Dependent variable

SOFA score was continuously and automatically calculated every four hours and recorded in the EMR. It was determined by an automated algorithm within the EMR system, assigning 0–4 points for each of 6 organ systems (neurologic, pulmonary, cardiovascular, renal, hepatic, hematologic), based on the most recent laboratory, respiratory, and nursing flowsheets, with increasing points associated with worse clinical function [8]. At YNHH, the algorithm used to calculate SOFA scores was developed by Epic support staff.

After development, a group of five physicians manually calculated SOFA scores for 50 patients across the ED, medicine floor, and ICUs to check the algorithm. Median disagreement between the manually calculated and automatically calculated scores was 0 points (IQR 0–1). For each piece of missing data in a flowsheet, the algorithm went back up to 72 hours to find a data point. If no data existed within 72 hours, the algorithm assigned 0 points (assuming a normal value). The algorithm theoretically accepted data from a prior admission, so long as the data were from within the time limit (72 hours). None of the 50 patients examined during the check of the algorithm had data pulled from a previous admission.

SOFA score was operationalized in two ways for this investigation. We examined peak overall SOFA score throughout the entire stay and peak 24-hour SOFA score. Peak overall SOFA score is an indicator of the severity of a patient’s clinical condition. We were especially interested in SOFA scores taken within the first 24 hours of the stay, as these scores would be used to determine the level of care and resources that patients receive. If minorities are initially sicker compared to white patients, as exhibited by higher peak 24-hour SOFA scores, then the use of SOFA scores in triage may lead to a systematic denial of care and resources to vulnerable populations. We also categorized 24-hour peak SOFA score into two categories, low <6 (n = 2190) and high ≥6 (n = 364), based on existing evidence that show poorer outcomes among patients with SOFA ≥6 [12, 16].

### Statistical analysis

We used chi-square tests and one-way ANOVA to assess differences in outcomes according to sociodemographic characteristics and differences in SOFA score across racial/ethnic patient groups. Linear and logistic regression was used to determine racial/ethnic differences in clinical outcomes and whether differences varied according to SOFA score level. Models were adjusted for age, sex, insurance, primary language, and Charlson comorbidity index.

## Results

The distribution of sample characteristics according to each outcome is presented in Table 1. Our final sample included 2,554 patients. Forty-three percent were white non-Hispanic, 25% were Black non-Hispanic, 26% were Hispanic of any race, 2% were of other non-Hispanic race, and 4% were of unknown race. The other non-Hispanic race category included forty-eight Asians, two American Indians/Alaskan Natives, one Native Hawaiian, and two patients documented as “other race.” Seventy nine percent of patients primarily spoke English, 18% spoke Spanish, and 3% spoke other languages. Most patients had Medicare (52%) followed by private insurance (20%) and Medicaid (20%).

**Table 1:** Clinical outcomes by sociodemographic characteristics of COVID+ patients, n = 2554.

Characteristic	Total n (%)*	Median Age (IQR, 25% - 75%)	Proportion in-hospital mortality (%)	p-value**	Proportion admitted to ICU (%)	p-value**
**Race/Ethnicity**				<0.001°		0.0362°
White Non-Hispanic	1086 (42.52)	75 (62–85)	158/1086 (14.55)		235/1086 (21.64)	
Black Non-Hispanic	637 (24.94)	62 (51–74)	70/637 (10.99)		169/637 (26.53)	
Hispanic, all races	674 (26.39)	54 ((39–66)	42/674 (6.23)		171/674 (25.37)	
Other Non-Hispanic	53 (2.08)	60 (44–75)	9/53 (16.98)		19/53 (35.85)	
Unknown	104 (4.07)	57 (36–70)	12/104 (11.54)		24/104 (23.08)	
**Age**				<0.001°		<0.001°
18–34	261 (10.22)		1/262 (0.38)		29/261 (11.11)	
35–44	228 (8.93)		1/228 (0.44)		48/228 (21.05)	
45–54	295 (11.55)		11/295 (3.73)		73/295 (24.75)	
55–64	484 (18.95)		32/484 (6.61)		149/484 (30.79)	
65–74	454 (17.78)		54/454 (11.89)		127/454 (27.97)	
75–84	435 (17.03)		94/435 (21.61)		133/435 (30.57)	
85+	397 (15.54)		98/397 (24.69)		59/397 (14.86)	
**Sex**				0.055		<0.001°
Women	1346 (52.70)	67 (49–82)	138/1346 (10.25)		261/1346 (19.39)	
Men	1208 (47.30)	63 (51–75)	153/1208 (12.67)		357/1208 (29.55)	
**Language preference**				0.001°		0.004°
English	2027 (79.37)	66 (52–81)	251/2027 (12.38)		469/2027 (23.14)	
Spanish	450 (17.62)	56 (43–68)	29/450 (17.65)		135/450 (30.00)	
Other	73 (2.86)	69 (55–81)	11/73 (15.07)		13/73 (17.81)	
**Insurance**				<0.001°		0.119
Private	507 (19.96)	53 (39–60)	18/507 (3.55)		100/507 (19.72)	
Medicaid	502 (19.76)	50 (34–60)	25/502 (4.98)		127/502 (25.30)	
Medicare	1316 (51.81)	77 (68–86)	230/1316 (17.48)		329/1316 (25.00)	
Medicare/Medicaid	22 (0.87)	77 (69–83)	6/22 (27.27)		7/22 (31.82)	
None	193 (7.60)	47 (38–60)	12/193 (6.22)		50/193 (25.91)	

*Sample sizes may not match due to missing data.

**Result of chi-square test.

°Significant at p = 0.05 level.

Table 1 presents patient characteristics by our main outcomes. A higher proportion of Spanish speaking patients were admitted to the ICU compared with English speakers (p = 0.004). The other non-Hispanic category had the highest mortality (17%), compared to 15% of white patients, 12% of unknown patients, 11% of Black patients, and 6% of Hispanic patients. Patients of other non-Hispanic race were also more likely to be admitted to the ICU (36%), while white patients were least likely to be admitted (22%). Spanish-speaking patients had higher in-hospital mortality compared to English-speaking patients (p = 0.001). Those with private insurance had lower in-hospital mortality compared to those with Medicaid, Medicare, dual, and the uninsured (p<0.001).

### Racial disparities in SOFA score

Table 2 presents clinical characteristics for COVID-positive patients by race/ethnicity. Overall, Black patients had higher SOFA scores, including mean 24-hour (p<0.001) and mean peak (p = 0.007) scores. Nineteen percent of Black patients had peak 24-hour SOFA scores ≥6 compared to 13% of white, 11% of Hispanic, 15% of patients of other non-Hispanic race, and 13% of patients of unknown race (p<0.001). Thirty one percent of Black patients had peak overall SOFA scores ≥6 compared to 32% of patients of other non-Hispanic race, 25% of white patients, 24% of Hispanic patients, and 21% of patients of unknown race. Black patients had significantly higher mean Charlson comorbidity index scores compared to all patients of other races. (p<0.001).

**Table 2:** Distribution of clinical characteristics in COVID+ patients by race/ethnicity, n = 2554.

Characteristic	N* (%)	White Non-Hispanic	Black Non-Hispanic	Hispanic, all races	Other Non-Hispanic	Unknown	p-value**
**24-Hour SOFA Score N (%)**							<0.001°
<6	2190 (85.75)	941 (86.65)	513 (80.53)	601 (89.17)	45 (84.91)	90 (86.54)	
≥6	364 (14.25)	145 (13.35)	124 (19.47)	73 (10.83)	8 (15.09)	14 (13.46)	
**Mean 24-Hour SOFA Score (SD)**	2554	2.51 (2.81)	2.97 (3.08)	2.15 (3.03)	2.43 (3.14)	2.27 (3.14)	<0.001°
**Peak SOFA Score N (%)**							0.020°
<6	1881 (73.65)	815 (75.05)	439 (68.92)	509 (75.52)	36 (67.92)	82 (78.85)	
≥6	673 (26.35)	271 (24.95)	198 (31.08)	165 (24.48)	17 (32.08)	22 (21.15)	
**Mean Peak SOFA Score (SD)**	2554	3.83 (3.62)	4.37 (4.12)	3.66 (4.31)	4.43 (4.75)	3.34 (3.90)	0.006°
**Mean 48 Hour Charlson Index (SD)**	2541	2.09 (2.30)	2.10 (2.38)	1.19 (1.99)	1.02 (1.69)	1.14 (1.91)	<0.001°

*Sample size may not match due to missing data.

** Result of chi-square test & one-way ANOVA.

°Significant at p = 0.05 level.

### In-hospital mortality

Overall and stratified logistic regression model results for in-hospital mortality are shown in Table 3. In the overall adjusted model, we controlled for race/ethnicity, age, sex, language preference, insurance, Charlson comorbidity index score, and SOFA score. In the stratified models, we controlled for all the variables listed except for SOFA score. Overall adjusted model results showed that patients of other non-Hispanic races had greater odds of in-hospital mortality compared to white patients (OR = 2.87, 95% confidence interval (CI) = 1.15, 7.13). SOFA-stratified model results showed that among those with SOFA <6, patients of other non-Hispanic races had greater odds of in-hospital mortality compared to white patients (OR = 3.55, 95% CI = 1.25, 10.08). Older age, male sex, and insurance status were associated with greater odds of in-hospital mortality.

**Table 3:** Factors associated with in-hospital mortality, stratified by 24-hour SOFA score***.

	Model 1 OR (95% CI): Overall		Model 2 OR (95% CI): SOFA <6		Model 3 OR (95% CI): SOFA ≥6	
Characteristic	*Unadjusted* **	*Adjusted (n = 2523)*	*Unadjusted* **	*Adjusted (n = 2164)*	*Unadjusted* **	*Adjusted (n = 359)*
**Race/Ethnicity**						
White Non-Hispanic	reference	reference	reference	reference	reference	reference
Black Non-Hispanic	0.73 (0.54, 0.98)*	1.09 (0.77, 1.54)	0.53 (0.36, 0.79)*	1.17 (0.76, 1.79)	0.96 (0.56, 1.64)	1.18 (0.65, 2.13)
Hispanic, all races	0.39 (0.27, 0.56)*	1.29 (0.69, 2.44)	0.33 (0.22, 0.51)*	1.27 (0.58, 2.75)	0.66 (0.34, 1.29)	1.68 (0.55, 5.13)
Other Non-Hispanic	1.29 (0.62, 2.71)	2.87 (1.15, 7.13)*	1.41 (0.61, 3.24)	3.55 (1.25, 10.08)*	0.85 (0.16, 4.36)	1.20 (0.50, 6.69)
Unknown	0.74 (0.40, 1.38)	1.69 (0.84, 3.41)	0.66 (0.31, 1.40)	1.74 (0.75, 4.03)	1.02 (0.30, 3.42)	1.82 (0.50, 6.69)
**Age**	1.06 (1.05, 1.07)*	1.07 (1.06, 1.08)*	1.07 (1.06, 1.09)*	1.07 (1.06, 1.09)*	1.05 (1.03, 1.07)*	1.06 (1.03, 1.08)*
**Sex**						
Women	reference	reference	reference	reference	reference	reference
Men	1.27 (0.99, 1.62)	1.57 (1.19, 2.08)*	1.29 (0.96, 1.73)	2.05 (1.48, 2.85)*	0.77 (0.48, 1.23)	1.03 (0.61, 1.74)
**Language preference**						
English	reference	reference	reference	reference	reference	reference
Spanish	0.49 (0.33, 0.73)*	0.57 (0.28, 1.19)	0.48 (0.29, 0.77)*	0.70 (0.29, 1.68)	0.57 (0.27, 1.18)	0.49 (0.13, 1.77)
Other	1.26 (0.65, 2.42)	0.98 (0.46, 2.09)	1.68 (0.84, 3.35)	1.12 (0.49, 2.57)	0.43 (0.05, 3.59)	0.42 (0.05, 3.88)
**Insurance**						
Private	reference	reference	reference	reference	reference	reference
Medicaid/Dual	1.71 (0.94, 3.09)	1.57 (0.82, 3.00)	2.07 (0.88, 4.88)	2.17 (0.88, 5.35)	1.05 (0.43, 2.55)	0.89 (0.34, 2.35)
Medicare	5.75 (3.52, 9.41)*	1.35 (0.77, 2.38)	9.67 (4.72, 19.84)*	2.23 (1.02, 4.88)	2.03 (0.96, 4.28)	0.74 (0.31, 1.79)
None	1.80 (0.85, 3.81)	2.27 (0.98, 5.29)	2.37 (0.85, 6.65)	2.89 (0.96, 8.67)*	1.29 (0.39, 4.34)	1.60 (0.41, 6.27)
**48 Hour Charlson Index**	1.17 (1.12, 1.23)*	1.07 (1.01, 1.13)*	1.20 (1.14, 1.27)*	1.10 (1.03, 1.17) *	1.08 (0.98, 1.18)	1.02 (0.92,1.13)
**24 Hour SOFA Score**	1.22 (1.18, 1.26)*	1.23 (1.18, 1.28)*				

*Significant at p = 0.05 level.

**Sample sizes for unadjusted models may be slightly different due to missing data.

***Overall adjusted model includes race/ethnicity, age, sex, language preference, insurance, Charlson index, and SOFA score. Stratified adjusted models include race/ethnicity, age, sex, language preference, insurance, and Charlson index.

### Admission to the ICU

Overall model results for ICU admission (Table 4) showed that after adjustment, patients of other non-Hispanic race had greater odds (OR = 2.78) of being admitted to the ICU compared to white patients (95% CI = 1.39, 5.54). There was no difference in odds of ICU admission among non-Hispanic Black and Hispanic patients compared with non-Hispanic white patients in the overall model or SOFA-stratified models. Other results showed that men (OR = 1.36, 95% CI = 1.11, 1.68) and patients whose primary language was Spanish (OR = 1.93, 95% CI = 1.23, 3.03) had greater odds of ICU admission.

**Table 4:** Factors associated with ICU admission, stratified by 24-hour SOFA score***.

Characteristic	Model 1 OR (95% CI): Overall		Model 2 OR (95% CI): SOFA <6		Model 3 OR (95% CI): SOFA ≥6	
	*Unadjusted* **	*Adjusted (n = 2523)*	*Unadjusted* **	*Adjusted (n = 2164)*	*Unadjusted* **	*Adjusted (n = 359)*
**Race/Ethnicity**						
**White Non-Hispanic**	reference	reference	reference	Reference	reference	reference
**Black Non-Hispanic**	1.31 (1.04, 1.64)*	1.16 (0.89, 1.52)	1.11 (0.83, 1.48)	1.20 (0.89, 1.63)	1.28 (0.78, 2.10)	1.47 (0.87, 2.49)
**Hispanic, all races**	1.23 (0.98, 1.54)	0.86 (0.57, 1.34)	1.34 (1.03, 1.75)*	0.89 (0.56, 1.42)	1.44 (0.80, 2.60)	0.79 (0.31, 2.02)
**Other Non-Hispanic**	2.04 (1.12, 3.70)*	2.78 (1.39, 5.54)*	1.87 (0.92, 3.81)	2.48 (1.17, 5.27)*	4.94 (0.59, 41.17)	3.75 (0.44, 31.96))
**Unknown**	1.10 (0.69, 1.77)	1.23 (0.71, 2.15)	0.94 (0.52, 1.69)	1.12 (0.61, 2.07)	2.59 (0.69, 9.68)	2.07 (0.54, 7.99)
**Age**	1.01 (1.00, 1.01)	1.00 (1.00, 1.01)	1.00 (1.00, 1.01)	1.01 (1.00, 1.02)	0.99 (0.98, 1.01)	1.00 (0.98, 1.02)
**Sex**						
**Women**	reference	reference	reference	Reference	reference	reference
**Men**	1.74 (1.45, 2.09)*	1.36 (1.11, 1.68)*	1.56 (1.25, 1.95)*	1.55 (1.23, 1.94)*	1.38 (0.89, 2.14)	1.35 (0.85, 2.15)
**Language preference**						
**English**	reference	reference	reference	Reference	reference	reference
**Spanish**	1.42 (1.13, 1.79)*	1.93 (1.23, 3.03)*	1.63 (1.25, 2.12)*	1.88 (1.16, 3.03)*	1.62 (0.86, 3.06)	2.47 (0.84, 7.23)
**Other**	0.72 (0.39, 1.32)	0.57 (0.29, 1.11)	0.81 (0.40, 1.66)	0.61 (0.28, 1.30)	0.81 (0.18, 3.68)	0.79 (0.17, 3.71)
**Insurance**						
**Private**	reference	reference	reference	Reference	reference	reference
**Medicaid/Dual**	1.40 (1.04, 1.88)*	1.31 (0.93, 1.84)	1.56 (1.09, 2.23)*	1.46 (1.00, 2.12)*	0.58 (0.28, 1.23)	0.61 (0.28, 1.34)
**Medicare**	1.36 (1.06, 1.75)*	1.06 (0.76, 1.48)	1.41 (1.03, 1.92)*	1.24 (0.85, 1.80)	0.63 (0.33, 1.22)	0.73 (0.34, 1.57)
**None**	1.42 (0.96, 2.10)	1.16 (0.72, 1.87)	1.65 (1.05, 2.62)*	1.27 (0.76, 2.10)	0.82 (0.28, 2.42)	0.59 (0.18, 1.94)
**48 Hour Charlson Index**	1.01 (0.97, 1.05)	0.97 (0.93, 1.02)	1.02 (0.97, 1.07)	1.02 (0.97, 1.07)	0.91 (0.83, 0.99)*	0.92 (0.84, 1.01)
**24 Hour SOFA Score**	1.39 (1.35, 1.44)*	1.40 (1.35, 1.45)*				

*Significant at p = 0.05 level.

**Sample sizes for unadjusted models may be slightly different due to missing data.

***Overall adjusted model includes race/ethnicity, age, sex, language preference, insurance, Charlson index, and SOFA score. Stratified adjusted models include race/ethnicity, age, sex, language preference, insurance, and Charlson index.

## Discussion

Our study aimed to determine whether the use of SOFA score as a triage tool for COVID-positive patients has the potential to exacerbate racial and ethnic disparities in clinical outcomes. We followed STROBE guidelines when reporting this work [29]. Black patients had higher mean peak overall and mean peak 24-hour SOFA scores. Black patients were also more likely to have a peak overall and a peak 24-hour SOFA score ≥6. However, we did not find significant differences in ICU admission and in-hospital mortality for Black patients in this study. The other non-Hispanic group, which was composed mostly of Asian patients, did have high rates of in-hospital mortality and ICU admission. However, because this was a small group that included patients of different backgrounds, we could not interpret these results.

Given the fact that Black patients nationwide have a higher burden of infection and mortality from COVID-19, it is worth considering why we found no substantial difference in ICU admission or in-hospital mortality between Black and white patients in our sample [2, 26, 30, 31]. Racial disparities in COVID-related mortality are highly variable from state to state [32]. In addition, a study from New York State found that higher mortality rates for Black community members were explained by higher rates of infection and hospitalization [33]. Because we examined in-hospital mortality and ICU admission in the context of EMR data, we were unable to look at disparities in infection or initial hospitalization. Despite this, our findings that Black COVID-19 patients had higher SOFA scores align with the overall literature that suggests a higher burden of morbidity of COVID-19 in Black patients [2, 26, 30, 31, 33].

The higher SOFA scores observed among Black patients demonstrate that they are sicker despite being younger on average compared to white patients (75 versus 62 median age). Though Black patients had higher 24-hour and peak SOFA score, this did not translate to statistically significant higher mortality or ICU admission compared to white patients. Therefore, if Black patients are sicker compared to white patients, as exhibited by higher SOFA scores, use of SOFA score to triage may lead to systematic denial of care and resources to Black patients, though clinical outcomes are similar. This would exacerbate existing health disparities.

State protocols have already recommended the use of SOFA to determine allocation of limited resources such as ventilators [33]. Some of these states primarily use the SOFA score while others combine SOFA score with measures like severe comorbidities or a modified Charlson comorbidity index. No matter the form of the SOFA score these triage protocols use, they all make use of a tool that has only been validated in sepsis patients and in settings with plenty of healthcare resources [12, 16]. The SOFA score was not developed for triage or created under an equity lens. This reflects a common problem: many well-established medical algorithms do not consider equity and lead to racial bias in resource allocation [34]. In one survey, 95% of hospitals with existing ventilator triage policies reported that their COVID ventilator triage protocols used some form of the SOFA score. The most commonly cited criteria for triage were reported as benefit, need, and conservation of resources—equity was not mentioned as a priority [35].

While individual triage protocols can and should be developed with an equity lens, these policies are too downstream to significantly impact the racial and ethnic disparities we see in COVID infections and mortality. For that, we need early, equitable, and widespread access to testing, fast and accurate contact tracing, and a well-funded and respected public health system. More broadly, we need to address the societal conditions that lead to disparate outcomes for Black, Indigenous, and other people of color through significant upstream interventions. These include the prevention of disparities in chronic disease, overcrowding and residential segregation, reparations and redistributive economic policies, and universal healthcare.

This work has some limitations. Firstly, this retrospective cohort study examined a single healthcare system early in the first surge of the COVID-19 pandemic. As such, these results can be generalized only to systems that are relatively similar to Yale-New Haven Health. Our analysis may have excluded some patients who were admitted on weekends, as SOFA scores were only calculated on weekdays. We conducted a sensitivity analysis using 48-hour rather than 24-hour SOFA score and obtained similar results to our original analysis. Finally, this study used documented race from the electronic health record. Previous work has shown that this data may be inaccurate and over-simplified for some patients [36]. There are ongoing efforts to improve the accuracy and specificity of these data to better serve patients and to support health equity research.

## Conclusions

Black individuals are more likely to get sick and die from COVID, and they are also more likely to suffer from the severe comorbidities that lead to worse SOFA scores and COVID outcomes; this important reality is not reflected in the use of SOFA scores or simple comorbidity calculations in triage protocols [2, 37, 38]. If used, these protocols have the potential to further exacerbate racial and ethnic disparities. Critical care clinicians should work to create triage protocols that will not exacerbate racial and ethnic disparities. These protocols may be downstream policies, but they are still important to develop for this pandemic and future crises. In a pandemic that is disproportionately harming Black, Latinx, Indigenous, and low-income communities, it is critical that healthcare systems develop and use equity-focused triage protocols and seek to change the racist systems that harm their patients’ health.
